# Heavy Metal–Resistant Plant Growth–Promoting *Citrobacter werkmanii* Strain WWN1 and *Enterobacter cloacae* Strain JWM6 Enhance Wheat (*Triticum aestivum* L.) Growth by Modulating Physiological Attributes and Some Key Antioxidants Under Multi-Metal Stress

**DOI:** 10.3389/fmicb.2022.815704

**Published:** 2022-05-06

**Authors:** Abdul Wahab Ajmal, Humaira Yasmin, Muhammad Nadeem Hassan, Naeem Khan, Basit Latief Jan, Saqib Mumtaz

**Affiliations:** ^1^Department of Biosciences, COMSATS University Islamabad, Islamabad, Pakistan; ^2^Department of Agronomy, Institute of Food and Agricultural Sciences, University of Florida, Gainesville, FL, United States; ^3^Department of Clinical Pharmacy, College of Pharmacy, King Saud University, Riyadh, Saudi Arabia

**Keywords:** heavy metals contamination, plant growth promoting bacteria, *Citrobacter* werkmanii and *Enterobacter* cloacae, wastewater irrigated agricultural soils, bioremediation and biofertilization, cadmium (Cd), lead (Pb), nickle (Ni)

## Abstract

Due to wastewater irrigation, heavy metal (HM) exposure of agricultural soils is a major limiting factor for crop productivity. Plant growth–promoting bacteria (PGPB) may lower the risk of HM toxicity and increase crop yield. In this context, we evaluated two HM-resistant PGPB strains, i.e., *Citrobacter werkmanii* strain WWN1 and *Enterobacter cloacae* strain JWM6 isolated from wastewater-irrigated agricultural soils, for their efficacy to mitigate HM (Cd, Ni, and Pb) stress in a pot experiment. Increasing concentrations (0, 50, 100, and 200 ppm) of each HM were used to challenge wheat plants. Heavy metal stress negatively affected wheat growth, biomass, and physiology. The plants under elevated HM concentration accumulated significantly higher amounts of heavy metals (HMs) in shoots and roots, resulting in increased oxidative stress, which was evident from increased malondialdehyde (MDA) content in roots and shoots. Moreover, alterations in antioxidants like superoxide dismutase (SOD), peroxidase (POD), ascorbate peroxidase (APX), and catalase (CAT) were observed in plants under HM stress. The severity of damage was more pronounced with rising HM concentration. However, inoculating wheat with *Citrobacter werkmanii* strain WWN1 and *Enterobacter cloacae* strain JWM6 (10^7^ CFU ml^–1^) improved plant shoot length (11–42%), root length (19–125%), fresh weight (41–143%), dry weight (65–179%), and chlorophyll a (14%-24%) and chlorophyll b content (2–24%) under HM stress. *Citrobacter werkmanii* strain WWN1 and *Enterobacter cloacae* strain JWM6 either alone or in co-inoculation enhanced the antioxidant enzyme activity, which may lower oxidative stress in plants. However, seeds treated with the bacterial consortium showed an overall better outcome in altering oxidative stress and decreasing HM accumulation in wheat shoot and root tissues. Fourier transform infrared spectroscopy indicated the changes induced by HMs in functional groups on the biomass surface that display effective removal of HMs from aqueous medium using PGPB. Thus, the studied bacterial strains may have adequate fertilization and remediation potential for wheat cultivated in wastewater-irrigated soils. However, molecular investigation of mechanisms adopted by these bacteria to alleviate HM stress in wheat is required to be conducted.

## Introduction

The agricultural sector is one of the most significant contributors to the world economy and serves as the basic livelihood of people in many countries, including Pakistan (Mishra et al., 2014). The importance of agriculture in providing food to an ever-increasing population can never be denied (Baldos and Hertel, 2014). However, regions around the globe practicing agriculture are facing numerous challenges, including shortage of freshwater and biotic and abiotic stresses under normal conditions (Hussain et al., 2018). Pakistan is among many other countries facing severe freshwater shortage. Due to shortage of freshwater and lack of treatment facilities, wastewater is disposed of by irrigating agricultural fields. This wastewater irrigation around industrial cities compensates for the deficiency of freshwater and supplies various nutrients necessary for plant growth (Khan and Bano, 2016). However, irrigation of agricultural fields with wastewater may alter the microbiological and physicochemical properties of soil and accumulate various biological and chemical contaminants in the land.

Among various pollutants, heavy metals (HMs) have become an alarming environmental hazard for the last few decades (Shoeva and Khlestkina, 2018). HMs enter soil via anthropogenic and geogenic sources such as industrial waste, fertilizers, sewage disposal, electroplating, and atmospheric deposition (Patel et al., 2018). HMs such as cadmium (Cd), chromium (Cr), mercury (Hg), nickel (Ni), arsenic (As), and lead (Pb) are thought to be highly toxic due to their bioaccumulative behavior and non-biodegradability (Taamalli et al., 2014). Earlier studies have highlighted that levels of Ni (30 mg/kg), Cd (6.1 mg/kg), and Pb (63.6 mg/kg) in wastewater-irrigated agricultural soils in different cities of Punjab, Pakistan, were above permissible limits (Ajmal et al., 2021; Iqbal et al., 2022). These HMs pose various environmental threats. For instance, even in small amounts, Cd in soils causes toxic effects on crops, such as reducing leaf photosynthetic efficiency, cell membrane lipid peroxidation, and inhibiting antioxidant enzymes (Rizwan et al., 2017; Ahanger et al., 2020; Kaya et al., 2020). Similarly, Pb is easily adsorbed in the soil and disturbs nutrient and plant water balance (Ashraf et al., 2015). At higher concentrations, Ni can inhibit cell division in meristematic root tissues and decrease photosynthesis and respiration (Bhalerao et al., 2015). These HMs obtained from soil enters human food chains via utilization of various cereal, legume, and vegetable crops (Kloke et al., 1984; Rizwan et al., 2016).

Among cereals, wheat (*Triticum aestivum* L.) serves as a staple food for above fifty percent of the world population. Hence, its demand is increasing with every coming day (Curtis and Halford, 2014). Meeting these increasing demands is a big question for policymakers and researchers (Shiferaw et al., 2013). Wheat can accumulate HMs in different parts like leaves, shoot, roots, and grains more than other cereal crops (Harris and Taylor, 2013). The transfer of HMs from soil to aerial parts of plants is dependent upon xylem and phloem loading (Page and Feller, 2005). So, it is crucial to minimize the uptake and translocation of HMs to edible parts of plants, which is an ultimate hazard for wheat-consuming populations (Keller et al., 2015).

In this regard, plant growth–promoting bacteria (PGPB) ameliorate plant productivity by limiting the adverse effects of biotic and abiotic stresses like pathogens, drought, salinity, and metal stress (Khanna et al., 2019a,b; Ahmad et al., 2021; Fahsi et al., 2021). The beneficial bacterial strains produce various metabolites, enzymes, and hormones that help to increase nutrient solubilization and stress alleviation. These mechanisms include siderophores, indole acetic acid (IAA), abscisic acid (ABA), ACC deaminase, and ethylene production (Mesa-Marín et al., 2018). Moreover, HM-tolerant PGPB increase plant root development and improve growth by raising the photosynthetic efficiency due to higher chlorophyll content and revamped PSII functionality (Giannakoula et al., 2021). Thus, HM-tolerant PGPB are an inexpensive, target-specific, and eco-friendly approach to cope with various biotic and abiotic stresses.

The resistance mechanisms against HMs induced by PGPB include biotransformation (change in the valence state of HMs) and bioaccumulation (HM accumulation inside the bacterial cell) (Mesa et al., 2015; Mallick et al., 2018). Therefore, PGPB can be exploited as biostimulants to increase plant growth by minimizing metal uptake by roots and restricting its transfer to aerial parts of plants (Sharaff et al., 2020). Moreover, to enhance HM resistance in plants, HM-tolerant PGPB are known to lessen the deterioration posed by reactive oxygen species (ROS) released by plants under HM stress by elevating the activity of several ROS-scavenging enzymes such as superoxide dismutase (SOD), peroxidase (POD), ascorbate reductase (APX), and catalase (CAT) (Ahmad et al., 2010, 2019; El-Meihy et al., 2019; Kohli et al., 2019).

Although HM-resistant PGPB are already reported, there are some limitations of these strains when used directly as biofertilizers (Borriss, 2011). Firstly, the soil is contaminated with several toxic HMs and chemicals; secondly, not all HM-resistant bacteria are plant growth promoters; and thirdly, not all bacterial strains can be active in all types of environmental conditions such as pH, temperature, humidity, and other soil properties. Therefore, isolation of potent multi-metal-resistant bacteria with PGP traits is in demand to increase crop productivity under metal stress conditions.

In this context, information is scanty about the potential of Cd-, Ni-, and Pb-resistant PGP *Citrobacter werkmanii* strain WWN1 and *Enterobacter cloacae* strain JWM6 to alleviate deleterious effects on wheat growing in Cd-, Ni-, and Pb-contaminated soils by modulating osmoregulation, photosynthetic machinery, and the antioxidant defense system. Therefore, this study aimed to reveal the potential of *Citrobacter werkmanii* strain WWN1 and *Enterobacter cloacae* strain JWM6 isolated from agricultural soils irrigated with wastewater to mitigate multi-metal toxicity in wheat. Moreover, the impact of PGPB to induce HM resistance in wheat by alteration in defense metabolism was also observed. This research may offer new ways to enhance crop yield by reducing HM toxicity to plants by the application of bacteria as biofertilizers.

## Materials and Methods

### Pot Experiment

A pot experiment was conducted in a greenhouse, located at COMSATS University Islamabad, Pakistan during November 2019 to March 2020. Seeds of wheat (PK-13) were obtained from the National Agricultural Research Center (NARC), Islamabad, Pakistan. *Citrobacter werkmanii* strain WWN1 (accession no.: MT941418) and *Enterobacter cloacae* strain JWM6 (accession no.: MT941425) were used to inoculate plants in this study. Both bacterial strains exhibited multi-HM (Cd, Ni, and Pb) resistance *in vitro* and were also able to remove these metals from the aqueous solution. *Citrobacter werkmanii* strain WWN1 and *Enterobacter cloacae* strain JWM6 removed 79, 87, and 43% and 78, 86, and 51% of Cd, Ni, and Pb, respectively, from 10 mg/L aqueous HMs solution. PGPB traits, i.e., phosphate, potassium, and zinc solubilization and protease and siderophore production, displayed by these strains were reported in a previous study (Ajmal et al., 2021).

The soil used in the pots contained total N = 0.05%, P = 16.2 mg/kg, Na = 4.3 meq/L, CaMg = 7.9 mq/L, Fe = 4.44 mg/kg, Zn = 0.72 mg/kg, EC = 1.3 dms^–1^, pH = 7.79, ESP = 2.3, and organic matter = 0.82%. The soil was passed through a 2-mm sieve, dried, and thoroughly mixed with HMs cocktail containing Cd as CdCl_2_⋅2H_2_O, Ni as NiSO_4_⋅6H_2_O, and Pb in the form of Pb(NO_3_)_2_ to obtain final concentrations of 50, 100, and 200 ppm for each of these three HMs and control with no HM addition. Three replicates were used for each treatment. Five kilograms of soil spiked with different concentrations of HMs was put in the plastic pots (22 × 17 cm) and was left to settle for 3 months (Li et al., 2019).

### Inoculum Preparation

A pure colony of each of *Citrobacter werkmanii* strain WWN1 and *Enterobacter cloacae* strain JWM6 was transferred to LB broth and incubated at 30 ± 1°C overnight to obtain respective bacterial culture. The bacterial cells were then centrifuged for 5 min at 6,000 × *g*. The resulting bacterial pellets were resuspended in sterilized distilled water (SDW). The bacterial cultures were maintained at 10^7^ CFU ml^–1^ by checking the optical density using a UV–visible spectrophotometer (Sudisha et al., 2006).

### Seed Sterilization and Inoculation

To surface sterilize, wheat seeds were soaked in 5% NaClO solution for 2 min and then again for 2 min in 70% ethanol. After that, seeds were rinsed with SDW thoroughly. Seeds were imbibed (12 h at room temperature) with bacterial cells (10^7^ ml^–1^) contained in 0.9% NaCl solution (Wang et al., 2016). Sterilized seeds were soaked in 0.9% NaCl solution and were sown in pots filled with HM-amended soil. Pots without added HMs and bacterial inoculation were treated as control. Pots were randomly kept in the greenhouse under natural conditions. The experiment was laid out as a completely randomized design (CRD) having three replicates for each treatment. Plants were uprooted after 120 days, and roots and shoots were parted for further analysis. A total of ten plants from each treatment were used for shoot and root length measurement and recording fresh and dry weights.

For various enzymatic analyses, the shoots and roots of sampled plants were separated and were stored at 4°C. For the estimation of bacterial colony-forming units (CFU), rhizospheric soil from each treatment was collected to check the viability of the applied bacterial inoculum. The CFU of rhizospheric soil was calculated by serial dilution and spread plate method (Humphris et al., 2005). Details of treatments used in the pot experiment are provided in Table 1.

**TABLE 1:** Details of the treatments used in the pot experiment.

Treatments	Details
Control	Non-inoculated without HM amendment
A	Inoculated with *Citrobacter werkmanii* strain WWN1 without HM amendment
B	Inoculated with *Enterobacter cloacae* strain JWM6 without HM amendment
AB	Inoculated with bacterial consortium without HM amendment
50, 100, 200 ppm	Inoculated with HMs at respective concentrations
50 ppm + A 50 ppm + B 50 ppm + AB	Inoculated with bacteria and HMs at respective concentrations
100 ppm + A 100 ppm + B 100 ppm + AB	Inoculated with bacteria and HMs at respective concentrations
200 ppm + A 200 ppm + B 200 ppm + AB	Inoculated with bacteria and HMs at respective concentrations

*HMs, Ni, Cd, and Pb.*

### Estimation of Photosynthetic Pigment Content

Chlorophyll a and chlorophyll b contents of wheat were assessed by adding 10 ml of dimethyl sulfoxide (DMSO) to fresh leaves (0.5 g) in Eppendorf tubes. The tubes were incubated at room temperature for 72 h or alternatively at 65°C in a water bath for 4 h. After incubation, the absorbance of the supernatant was checked at 665-nm and 645-nm wavelengths. Chlorophyll a and chlorophyll b were estimated by the following formula (Shoaf and Lium, 1976):

Chl a mg/g = [12.7 (OD 663) – 2.69 (OD645)] × V/1000 × W

Chl b mg/g = [22.9 (OD645) – 4.68 (OD663)] × V/1000 × W

where

V, volume of extract.

W, weight of sample.

### Measurement of Malondialdehyde Content

To determine the intensity of oxidative damage on the membrane due to HM toxicity, malondialdehyde formation in roots and shoots of wheat was detected as an indication of lipid peroxidation. Briefly, 10 ml (0.1%) of trichloroacetic acid (TCA) was used to homogenize 0.5 g fresh plant tissues, and centrifugation was done at 12,000 *g*. Four milliliters of TCA containing 5% thiobarbituric acid (TBA) was added to 1 ml of the supernatant. Heating of the mixture was carried out at 95°C for 25 min, and then immediate cooling was done on ice. Centrifugation of the reactant mixture was carried out at 12,000 *g* for 10 min, and the absorbance of the supernatant was recorded at 532 and 660 nm (Buege and Aust, 1978).

### Measurement of Proline Content

Proline content was assessed using acidic ninhydrin (Troll and Lindsley, 1955) with slight modifications. Aqueous sulfosalicylic acid (5 ml) was used to homogenize half a gram of fresh leaves. The reaction mixture was boiled for 10 min and was then allowed to cool. Two milliliters of glacial acetic acid and 4 ml of acidic ninhydrin were added to the supernatant and were placed in a boiling water bath. The reaction mixture was then cooled to room temperature followed by addition of 4 ml of toluene. The mixture was vortexed and allowed to settle. After that, the supernatant was segregated and absorbance was recorded at 520 nm. The proline content was determined by comparing the recorded values of absorbance with a standard curve of known concentration of L-proline and was expressed as l μg/g FW of leaf tissue.

### Determination of Antioxidative Enzyme Activities

#### Preparation of Enzyme Extracts

For the preparation of crude enzyme extract, 0.5 g fresh wheat shoots and roots were taken separately and rinsed with distilled water (DW). Homogenization of samples was carried out on ice in 5 ml sodium phosphate buffer (pH 7.8). After that, samples were centrifuged at 5000 *g* for 20 min at 4°C. The crude enzyme extract was denied light exposure by covering it with aluminum foil and was preserved at 4°C for various enzymatic assays (Azmat et al., 2020).

#### Superoxide Dismutase Activity

Photoreduction of nitroblue tetrazolium (NBT) was followed to check the SOD activity of the extract (Beauchamp and Fridovich, 1971). The reaction mixture included 50 mM phosphate buffer of pH 7.8, 13 mM L-methionine, 0.1 mM EDTA, 75 μM NBT, 8 μM riboflavin, and 100 μL of crude enzyme extract. Riboflavin was added lastly, and the reaction was initiated by exposing the mixture to 20-W fluorescent lamps. The reaction was then terminated by removing the mixture from the light source after 15 min. The photoreduction of NBT was noted at 560 nm using a spectrometer.

#### Catalase Activity

Catalase activity was measured by using the method of Kumar et al. (2010) with slight modifications. The absorbance of the reaction mixture with 100 μL of 300 mM H_2_O_2_, 2.8 ml of dilute 50 mM phosphate buffer (pH 7.0), and 100 μL of crude enzyme extract was measured at 240 nm.

#### Peroxidase Activity

4-Methylcatechol, which causes oxidation when mixed with H_2_O_2_, was used as a substrate to determine the POD activity of crude extract (Gorin and Heidema, 1976). The reaction mixture (3 ml) was put together by adding 100 mM sodium phosphate buffer (pH 7.0), 5 mM H_2_O_2_, 5 mM 4-methylcatechol, and 500 μl of crude extract, and the absorbance was recorded at 420 nm.

#### Ascorbate Peroxidase Activity

Ascorbate peroxidase activity was recorded by monitoring the rate of ascorbate oxidation at 290 nm (Yoshimura et al., 2000). The reaction mixture was composed of 25 mM phosphate buffer (pH 7), 1 mM H_2_O_2_, 0.5 mM ascorbic acid, and 100 μl of crude enzyme extract.

### Determination of Heavy Metals in Plant Tissues

The total Cd, Ni, and Pb concentration in plant roots and shoots was assessed by the method described by Karstensen et al. (1998). HM accumulation in plant tissues was determined by the wet mineralization method (Lozano-Rodríguez et al., 1995). Briefly, plant tissues were washed with deionized distilled water (DDW) and dried at 80°C until a constant weight was achieved. After this, 0.25 g of each plant sample was finely ground. Three milliliters of nitric acid (65% v/v) and 2 ml of hydrogen peroxide (35% v/v) were added, and the mixture was autoclaved. After cooling, the solution volume was raised up to 50 ml using DW, and HMs were detected using atomic absorption spectroscopy.

### Fourier Transform Infrared Spectroscopy Analysis of Heavy Metals Compounded to Bacterial Cells

Bacterial strains were inoculated in nutrient media spiked with 200 ppm of Pb, Ni, and Cd for 48 h and no HM-supplemented medium was treated as control. The samples were centrifuged at 4000 g for 15 min at 4°C. The resulting bacterial pellets were rinsed three times with sterilized deionized water, lyophilized, and ground in a freeze dryer. The dried bacterial biomass was used for Fourier transform infrared spectroscopy (FTIR) (Rodríguez-Sánchez et al., 2017).

### Statistical Analysis

One-way (ANOVA) suited for CRD was applied to statistically analyze the samples. The mean values of the replicates were compared, and the correlation coefficient was calculated using Statistix 8.1. The significance of the difference among the treatments was measured using the least significant difference (LSD) at significance level *P* < 0.05 (Steel and Torrie, 1960). A correlation matrix heatmap was created using Origin 2020b. Principal coordinate analysis was carried out using PRIMER (Plymouth Routines in Multivariate Ecological Research), version 6.1.12, Primer-E Ltd, Plymouth, United Kingdom (Clarke and Warwick, 2001).

## Results

### Germination Percentage

All the samples without HM (Cd, Ni, and Pb) stress whether uninoculated or inoculated with bacteria showed no significant effect on germination percentage. Similarly, no significant difference was recorded in germination percentage in the presence of 50 ppm, 100 ppm, or 200 pm of HM stress when plants were inoculated with bacteria compared to non-inoculated plants.

### Colony-Forming Units of Rhizospheric Soil (CFU g^–1^)

**FIGURE 1:**
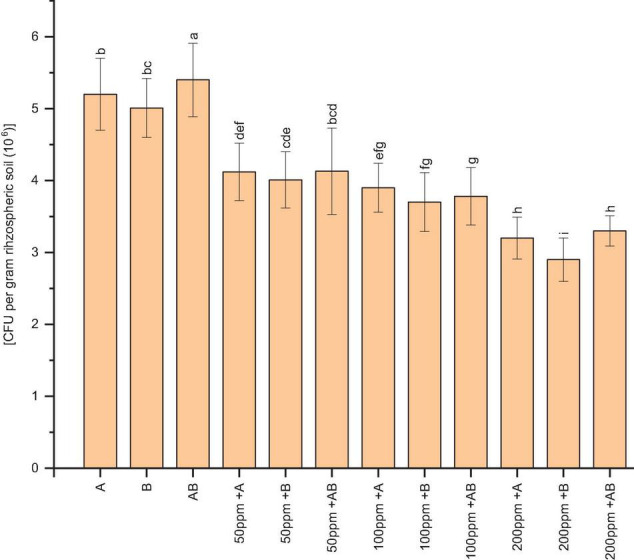
Bacterial colonization [CFU per gram rhizospheric soil (×10^6^)] in the rhizosphere of wheat under control and heavy metal (Cd, Ni, Pb) stress conditions. All the values are the mean of three replicates. Bars represent the standard error of means. Different letters above the bars indicate statistically significant difference between treatments at *P* ≤ 0.05. A, *Citrobacter werkmanii* strain WWN1; B, *Enterobacter cloacae* strain JWM6; A + B, *Citrobacter* + *Enterobacter* consortium; 50 ppm, 50 ppm of Cd, Ni, and Pb; 100 ppm, 100 ppm of Cd, Ni, and Pb; 200 ppm, 200 ppm of Cd, Ni, and Pb.

The bacterial population of wheat rhizospheric soil was significantly reduced under HM stress. At 0 and 50 ppm of HMs, the CFU g^–1^ of rhizospheric soil was recorded as 5.4 × 10^6^ and 4.13 × 10^6^, respectively, after plants had been inoculated with the bacterial consortium. Under 100 ppm of HMs, *Citrobacter werkmanii* strain WWN1 showed the highest CFU g^–1^ of rhizospheric soil (3.9 × 10^6^). In case of 200 ppm HM concentration, the bacterial consortium had the highest CFU g^–1^ of rhizospheric soil, i.e., 3.4 × 10^6^ (Figure 1).

### Effect of Heavy Metal Concentration and the Bacterial Inoculation on Wheat Growth

Heavy metal stress had an adverse effect on the overall growth of plants. With rising HM concentration in soil, the shoot and root length decreased gradually. Under no HM stress, the shoot length ranged from 48 to 69 cm. The maximum increase in shoot length (41%) was exhibited by plants inoculated with *Citrobacter werkmanii* strain WWN1 compared to the control. At 50 ppm HM concentration, the range of shoot length was 45–59 cm. *Citrobacter werkmanii* strain WWN1 and *Enterobacter cloacae* strain JWM6 alone showed an increase of 33 and 34%, respectively, compared to uninoculated plants. When bacteria were applied in the consortium, an increase of 32% was observed for shoot length. Under 100 ppm, the shoot length ranged from 50 to 66 cm. A maximum increase of 40% (66 cm) was displayed by plants inoculated with the bacterial consortium. In case of 200 ppm, the shoot length of wheat ranged from 42 to 60 cm. A maximum increase of 42% was again observed under combined application of *Citrobacter werkmanii* strain WWN1 and *Enterobacter cloacae* strain JWM6 compared to uninoculated plants.

**FIGURE 2:**
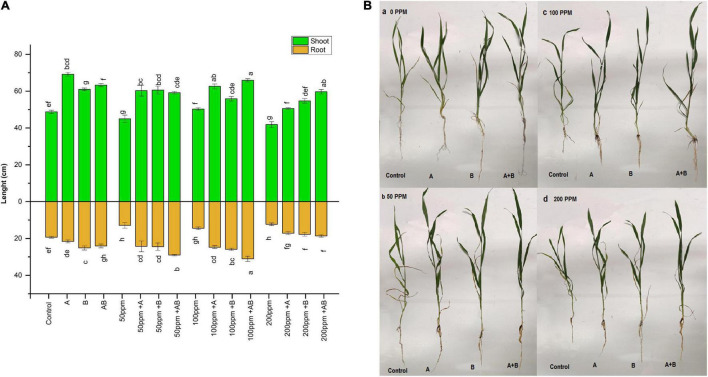
**(A)** Effect of heavy metal–resistant plant growth–promoting bacteria on the length of shoots and roots of wheat (*Triticum aestivum*) under control and heavy metal (Cd, Ni, Pb) stress conditions. Values are mean of three replicates. Bars represent the standard error of means. Different letters above the bars indicate statistically significant difference between treatments at *P* ≤ 0.05. Details of treatments are the same as those in Figure 1. **(B)** Effect of heavy metal (a) 0 ppm, (b) 50 ppm, (c) 100 ppm, and (d) 200 ppm resistant plant growth–promoting bacteria on the growth of wheat (*Triticum aestivum*) under control and heavy metal (Cd, Ni, Pb) stress conditions. Details of treatments are the same as those in Figure 1.

A similar trend was noticed in roots of plants under HM stress. At 0 ppm of HMs, the root length ranged from 19.3 to 25.1 cm. A maximum increase of 30% (25.1 cm) was observed in plants inoculated with *Enterobacter cloacae* strain JWM6. At 50 and 100 ppm, root lengths ranged from 12.8 to 29 cm and 14.4 to 31 cm, respectively. Under both of these concentrations, the bacterial consortium showed the maximum increase in root length where an increase of 125% (29 cm) and 114% (31 cm) was observed in plants inoculated with consortium as compared to uninoculated plants. Under 200 ppm of HM stress, wheat root length was measured from 12.2 to 18.5 cm. *Citrobacter werkmanii* strain WWN1 and *Enterobacter cloacae* strain JWM6 inoculated plants showed an increase of 39 and 44% in plant root length, respectively, while an increase of 51% (18.5) was observed when plants were inoculated with the bacterial consortium. Overall, under all HM concentrations, the bacterial consortium showed better improvement in shoot and root length (Figures 2A,B).

### Effect of Heavy Metal Concentration and the Bacterial Inoculation on Wheat Biomass

**FIGURE 3:**
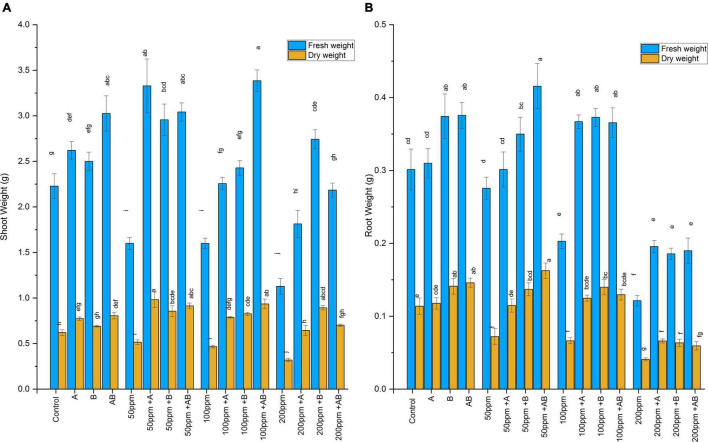
**(A)** Effect of heavy metal–resistant plant growth–promoting bacteria on the fresh and dry weights of the shoots of wheat (*Triticum aestivum*) under control and heavy metal (Cd, Ni, Pb) stress conditions. Values are the mean of three replicates. Bars represent the standard error of means. Different letters above the bars indicate statistically significant difference between treatments at *P* ≤ 0.05. Details of treatments are the same as those in Figure 1. **(B)** Effect of heavy metal–resistant plant growth–promoting bacteria on the fresh and dry weights of the roots of wheat (*Triticum aestivum*) under control and heavy metal (Cd, Ni, Pb) stress conditions. Values are the mean of three replicates. Bars represent the standard error of means. Different letters above the bars indicate statistically significant difference between treatments at *P* ≤ 0.05. Details of treatments are the same as those in Figure 1.

The wet and dry weights of plant shoots and roots diminished with rising HM concentration. However, bacterial treatment significantly improved shoot and root biomass under HM stress. At 0 ppm of HMs, the shoot fresh weight ranged from 2.2 to 3.05 g and the dry weight ranged from 0.62 to 0.8 g. A maximum rise of 38 and 29% in shoot fresh and dry weights was detected under the bacterial consortium. At 50 ppm, the fresh weight was recorded from 1.6 to 3.3 g and the dry weight ranged from 0.51 to 0.98 g. The maximum increase in shoot biomass was observed in plants after *Citrobacter werkmanii* strain WWN1 inoculation where an increase of 108% in shoot fresh weight and 90% in shoot dry weight was detected as compared to the uninoculated plants. In case of 100 ppm of HMs, the fresh weight recorded was from 1.6 to 3.38 g and the dry weight ranged from 0.46 to 0.93 g. The greatest increase of 112% in fresh weight and 100% in dry weight was observed in plants inoculated with the bacterial consortium. At 200 ppm HM stress, the fresh shoot weight ranged from 1.12 to 2.7 g and the dry weight ranged from 0.32 to 0.89 g. *Enterobacter cloacae* strain JWM6 inoculated plants showed the highest increase in fresh shoot weight (143%) and dry weight (117%) as compared to uninoculated plants (Figure 3A).

Heavy metals also had a negative impact on root biomass. However, inoculating plants with bacteria significantly enhanced root fresh and dry weights. Under 0 ppm of HMs, the root fresh weight ranged from 0.3 to 0.38 g and the dry weight was recorded in the range of 0.11 to 0.15 g. *Enterobacter cloacae* strain JWM6 and the bacterial consortium increased the root fresh weight by 24% and the root dry weight by 24 and 28%, respectively, when compared with the control. In case of 50 ppm of HMs, the fresh root weight ranged from 0.27 to 0.41 g and the dry weight was in the range of 0.07 to 0.16 g. A maximum rise of 51% in fresh weight and 125% in dry weight was observed when plants were applied with the bacterial consortium. At 100 ppm, root fresh and dry weights ranged from 0.2 to 0.37 g and 0.06 to 0.14 g, respectively. An increase of 84 and 110% in root fresh and dry weight was observed in plants inoculated with *Enterobacter cloacae* strain JWM6 compared to untreated plants. In case of 200 ppm, the fresh weight recorded was within 0.12–0.2 g and the dry weight was observed in the range of 0.04–0.06 g. An increase of 61% in both fresh and dry weights was observed in plants treated with *Citrobacter werkmanii* strain WWN1 (Figure 3B).

### Effect of Heavy Metals and the Bacteria on Photosynthetic Pigments

**FIGURE 4:**
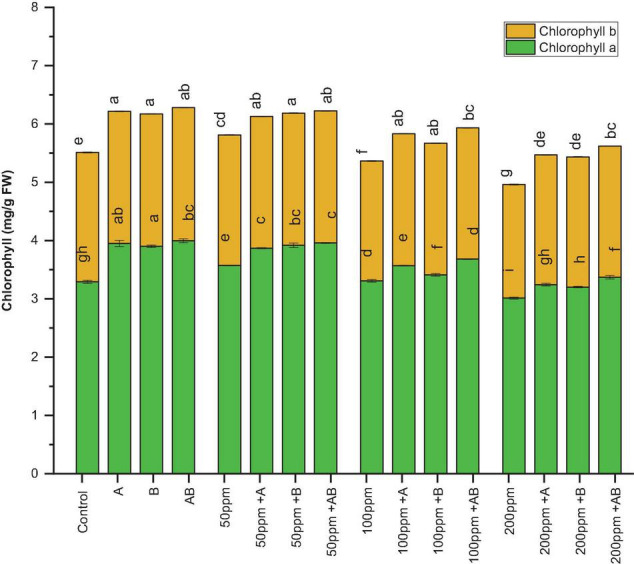
Effect of heavy metal–resistant plant growth–promoting bacteria on chlorophyll a and chlorophyll b of wheat (*Triticum aestivum*) under control and heavy metal (Cd, Ni, Pb) stress conditions. Values are the mean of three replicates. Bars represent the standard error of means. Different letters above the bars indicate statistically significant difference between treatments at *P* ≤ 0.05. Details of treatments are the same as those in Figure 1.

The leaf chlorophyll content declined with an increase in HM concentration. However, bacterial inoculation significantly amplified the chlorophyll content under HM stress. Under 0 ppm of HMs, the chlorophyll a content ranged from 3.7 to 4.1 mg/g FW. Both *Citrobacter werkmanii* strain WWN1 and *Enterobacter cloacae* strain JWM6 increased the chlorophyll a content by 17 and 15%, respectively, while an increase of 21% (4.1 mg/g FW) was observed in case of plants under the inoculation of the bacterial consortium. Similarly, under HM stress, plants treated with PGPB showed a significant increase in chlorophyll a content as compared to uninoculated plants. At 50 ppm HM concentration, the chlorophyll a content ranged from 3.1 to 3.9 mg/g FW. Here *Citrobacter werkmanii* strain WWN1 increased chlorophyll a by 22%, *Enterobacter cloacae* strain JWM6 increased the chlorophyll a content by 24% (3.9 mg/g FW), and the bacterial consortium increased the chlorophyll a content by 23% in comparison to uninoculated plants. Under 100 and 200 ppm, the chlorophyll a content ranged from 2.98 to 3.68 mg/g FW and 2.76 to 3.36 mg/g FW, respectively, and a maximum increase of 23% (3.68 mg/g FW) and 21% (3.36 mg/g FW) was noticed in plants treated with the bacterial consortium when compared with uninoculated plants (Figure 4).

Similarly, the chlorophyll b content ranged from 2.21 to 2.27 mg/g FW without HM stress and *Citrobacter werkmanii* strain WWN1 and *Enterobacter cloacae* strain JWM6 increased the chlorophyll b content by 2.1 and 2.3% respectively and their consortium increased the chlorophyll b content by 1.9% (2.26 mg/g FW) under 0 ppm of HMs. Under HM stress, plants inoculated with PGPB showed a significant rise in chlorophyll b content. For example, under 50 ppm HM stress, the chlorophyll b content ranged from 2.05 to 2.28 mg/g FW. Here *Citrobacter werkmanii* strain WWN1 and *Enterobacter cloacae* strain JWM6 alone increased the chlorophyll b content by 9.8 and 10%, respectively, and an increase of 11% (2.28 mg/g FW) was observed in plants treated with the bacterial consortium in comparison to untreated plants. In case of 100 ppm, *Citrobacter werkmanii* strain WWN1 and *Enterobacter cloacae* strain JWM6 increased the chlorophyll b content by 18 and 17%, respectively, and by 17% (2.25 mg/g FW) when these strains were applied in consortium. Under 200 ppm, the chlorophyll b content ranged from 1.81 to 2.25 mg/g FW and an increase of 22 and 23% was observed in plants inoculated with *Citrobacter werkmanii* strain WWN1 and *Enterobacter cloacae* strain JWM6, respectively. When applied in consortium, these two strains augmented the chlorophyll b content by 24% (2.25 mg/g FW) compared to uninoculated plants (Figure 4).

### Effect of Heavy Metal Stress and the Bacterial Inoculation on Malondialdehyde Content

The MDA content of both shoots and roots of plants increased with an increase in the HM concentration. However, bacterial inoculation lowered the MDA content significantly. In pots added with 0 and 50 ppm HMs, root MDA ranged from 2.1 to 3.9 nmol g^–1^ FW and 7.7 to 2.4 nmol g^–1^ FW, respectively. A maximum decline of 44% (2.1 nmol g^–1^ FW) and 68% (2.4 nmol g^–1^ FW) was observed in plant roots treated with *Enterobacter cloacae* strain JWM6 under 0 and 50 ppm of HMs, respectively, compared to uninoculated plants. In case of 100 ppm, root MDA ranged from 9.4 to 3.5 nmol g^–1^ FW and a decline of 61 and 51% was observed in plants inoculated with *Citrobacter werkmanii* strain WWN1 and *Enterobacter cloacae* strain JWM6, respectively, while both of these strains together reduced root MDA by 62% (3.5 nmol g^–1^ FW) in comparison to uninoculated plants. At 200 ppm, the shoot MDA content measured was between 14.63 and 6.6 nmol g^–1^ FW. A maximum decrease of 54% was noticed in plants applied with the bacterial consortium as compared to uninoculated plants.

**FIGURE 5:**
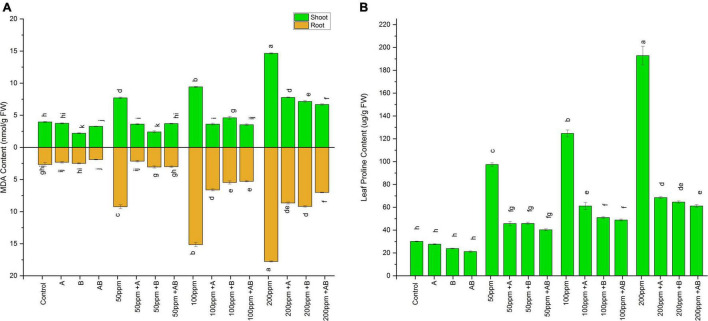
Effect of heavy metal–resistant plant growth–promoting bacteria on **(A)** MDA and **(B)** proline activity of wheat (*Triticum aestivum* L.) in plants under control and heavy metal (Cd, Ni, Pb) stress conditions. Values are the mean of three replicates. Bars represent the standard error of means. Different letters above the bars indicate statistically significant difference between treatments at *P* ≤ 0.05. Details of treatments are the same as those in Figure 1.

A similar trend was observed in wheat shoots, where the MDA content increased with rising HM concentrations. However, bacterial inoculation significantly dropped the MDA content as compared to the uninoculated plants. In shoots under 0 ppm of HMs, the MDA content ranged from 1.89 to 2.66 nmol g^–1^ FW. *Citrobacter werkmanii* strain WWN1 and *Enterobacter cloacae* strain JWM6 decreased the MDA content by 12 and 6%, respectively, while a decline of 29% (1.89 nmol g^–1^ FW) was recorded in plants inoculated with the bacterial consortium. Under 50 ppm of HMs in soil, the MDA content ranged from 2.1 to 9.42 nmol g^–1^ FW. *Citrobacter werkmanii* strain WWN1 and *Enterobacter cloacae* strain JWM6 inoculated plants showed a reduction of 76% (2.1 nmol g^–1^ FW) and 66% in MDA level, respectively, when compared with uninoculated plants. In case of 100 ppm HM stress, MDA ranged from 5.29 to 15.14 nmol g^–1^ FW, whereas *Citrobacter werkmanii* strain WWN1 decreased shoot MDA content by 56% and *Enterobacter cloacae* strain JWM6 lowered MDA content by 63%. When these bacteria were applied in the form of consortium, an overall decrease of 65% (5.29 nmol g^–1^ FW) was observed compared to uninoculated plants. Under 200 ppm of HM stress, shoot MDA ranged from 17.7 to 7.01 nmol g^–1^ FW. *Citrobacter werkmanii* strain WWN1 and *Enterobacter cloacae* strain JWM6 alone decreased the shoot MDA content by 51 and 49%, respectively, while when applied in consortium, a decline of 60% (7.01 nmol g^–1^ FW) was observed compared to uninoculated plants. Overall, the bacterial consortium proved to be better in reducing the MDA content under higher HM stress than single bacterial treatment (Figure 5A).

### Effect of Heavy Metal Stress and the Bacteria on Proline Content

Like MDA, the proline content also escalated with rising HM stress. However, inoculation of PGPB significantly lowered the leaf proline content. Without HM stress, the proline content measured was in the range of 21–30 μg/g FW. A maximum reduction of 30% (21 μg/g FW) in proline content was measured in plants inoculated with the bacterial consortium. Under 50 ppm and 200 ppm, proline ranged from 40 to 97 μg/g FW and 61 to 192 μg/g FW respectively. The bacterial consortium showed a maximum decline of 58% at 50 ppm and 68% at 200 ppm as compared to non-inoculated plants. However, in the case of 100 ppm, the proline level ranged from 49 to 124 μg/g FW. Here *Citrobacter werkmanii* strain WWN1 and *Enterobacter cloacae* strain JWM6 lowered the leaf proline content by 50 and 59%, respectively. When applied in a consortium, bacteria reduced the leaf proline content by 61% (49 μg/g FW) compared to uninoculated plants. Thus, under all three concentrations of HMs, the bacterial consortium dropped the proline content more than individual bacterial strains (Figure 5B).

### Effect of Heavy Metal Exposure and the Bacterial Inoculation on Antioxidative Enzyme Activity of Wheat

#### Superoxide Dismutase Activity

The SOD activity in wheat shoots and roots declined with rising HM concentrations. However, bacterial treatment significantly boosted the shoot as well as root SOD content.

For shoots, at 0 ppm of HMs, the SOD enzyme level ranged from 14.5 to 20.7 EU mg^–1^ protein. An increase of 43% (20.7 EU mg^–1^ protein) and 23% was observed in plants treated with *Citrobacter werkmanii* strain WWN1 and *Enterobacter cloacae* strain JWM6, respectively, while bacterial consortia enhanced shoot SOD by 30% when compared with the control. In case of 50 ppm HM stress, the SOD content ranged from 13 to 29 EU mg^–1^ protein. A maximum rise of 111% (27.5 EU mg^–1^ protein) was displayed by plants applied with *Enterobacter cloacae* strain JWM6. Under 100 ppm HM concentration, the level of SOD was recorded in the range from 6 to 26 EU mg^–1^ protein. *Citrobacter werkmanii* strain WWN1 and *Enterobacter cloacae* strain JWM6 increased SOD by 293 and 322%, respectively, while plants treated with the bacterial consortium showed an increase of 332% (26 EU mg^–1^ protein) when compared to untreated plants. At 200 ppm, the shoot SOD content was in the range of 5.6–16 EU mg^–1^ protein. Both *Citrobacter werkmanii* strain WWN1 and *Enterobacter cloacae* strain JWM6 alone boosted SOD activity in shoot by 182 and 181%, respectively, and the consortium of these two strains elevated the shoot SOD content by 103% (11.5 EU mg^–1^ protein).

**FIGURE 6:**
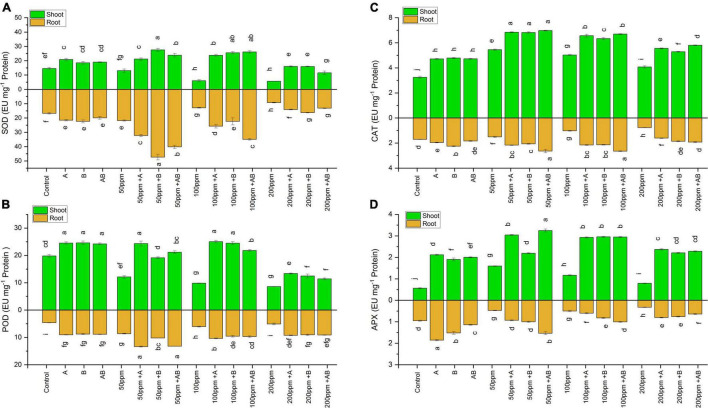
Effect of heavy metal–resistant plant growth–promoting bacteria on **(A)** SOD, **(B)** POD, **(C)** CAT, and **(D)** APX activity of wheat (*Triticum aestivum* L.) in plants under control and heavy metal (Cd, Ni, Pb) stress conditions. Values are the mean of three replicates. Bars represent the standard error of means. Different letters above bars the indicate statistically significant difference between treatments at *P* ≤ 0.05. Details of treatments are the same as those in Figure 1.

Similarly, in roots, in the absence of HM stress, the SOD content ranged from 16 to 22.4 EU mg^–1^ protein. An increase of 28% was recorded in plants inoculated with *Citrobacter werkmanii* strain WWN1, and a rise of 33% (22.3 EU mg^–1^ protein) was seen in plants treated with *Enterobacter cloacae* strain JWM6, while the bacterial consortium increased root SOD by 18% in comparison to untreated plants. At 50 ppm of HMs, the SOD content ranged from 21.7 to 47 EU mg^–1^ protein. A maximum increase of 117% (47 EU mg^–1^ protein) was observed in plant roots inoculated with *Enterobacter cloacae* strain JWM6. In case of 100 ppm, root SOD was in the range of 12.8–34.8 EU mg^–1^ protein. Plants treated with the bacterial consortium showed the highest increase of 170% (34.8 EU mg^–1^ protein) in root SOD when compared with non-treated plants. Under 200 ppm of HMs, root SOD ranged from 9.2 to 16.2 EU mg^–1^ protein. Plants applied with *Citrobacter werkmanii* strain WWN1 showed an increase of 75% (16.2 EU mg^–1^ protein), while *Enterobacter cloacae* strain JWM6 raised SOD by 51%, and their consortium enhanced SOD activity by 42%. Overall, the SOD content in plant roots was greater than that in shoots (Figure 6A).

#### Peroxidase Activity

The peroxidase activity in the shoots and roots of wheat plants was amplified with an increase in HM concentration, and treatment with PGPB further enhanced POD in the shoots and roots of wheat in both the absence and presence of HMs. In case of no HMs, shoot POD ranged from 19.8 to 24.5 EU mg^–1^ protein. *Citrobacter werkmanii* strain WWN1 and *Enterobacter cloacae* strain JWM6 were able to increase shoot POD activity by 23 and 24%, respectively, while the bacterial consortium showed an increase of 22% compared to non-treated plants. In the case of 50 and 100 ppm, POD occured between 12 and 24.6 EU mg^–1^ protein and 9.8 and 21.8 EU mg^–1^ protein, respectively, and at 200 ppm, POD activity ranged from 8.5 to 13.3 EU mg^–1^ protein. Among all these three treatments, *Citrobacter werkmanii* strain WWN1 showed the maximum rise in shoot POD activity with 100% increase at 50 ppm, 154% increase at 100 ppm, and 56% rise at 200 ppm as compared to uninoculated plants.

In case of roots, a similar trend was observed where root POD increased with an increase in HM concentration and bacterial inoculation further enhanced root POD in the absence and presence of HMs. Under 0 ppm of HMs, root POD ranged from 4.6 to 9 EU mg^–1^ protein. *Citrobacter werkmanii* strain WWN1 and *Enterobacter cloacae* strain JWM6 inoculated plants showed an increase of 96% (9 EU mg^–1^ protein) and 90%, respectively, and the bacterial consortium enhanced root POD by 92% as compared to the control. In case of 50 ppm, root POD ranged from 8.6 to 13.35 EU mg^–1^ protein. An increase of 54 and 19% was observed in plants inoculated with *Citrobacter werkmanii* strain WWN1 and *Enterobacter cloacae* strain JWM6, respectively, while the bacterial consortium increased POD activity by 53% (13.32 EU mg^–1^ protein) as compared to their respective controls. At 100 ppm, the root POD content ranged from 6.1 to 10.3 EU mg^–1^ protein. A maximum increase of 71% (10.3 EU mg^–1^ protein) was observed in plants inoculated with *Citrobacter werkmanii* strain WWN1, while at 200 ppm of HMs, POD ranged from 5 to 9.2 EU mg^–1^ protein. PGPB-inoculated plants showed an increase in POD activity, i.e., 82% (9.2 EU mg^–1^ protein) increase by *Citrobacter werkmanii* strain WWN1, 78% by *Enterobacter cloacae* strain JWM6, and 80% increase in POD by the bacterial consortium compared to the respective control (Figure 6B).

#### Catalase Activity

The catalase activity in wheat shoots and roots was significantly higher under HM stress than no HM stress, and the bacterial inoculation further increased the CAT activity under HM stress. At 0 ppm of HMs, shoot CAT activity ranged from 3.2 to 4.8 EU mg^–1^ protein. An increase of 45 and 47% (4.8 EU mg^–1^ protein) was observed in plants inoculated with *Citrobacter werkmanii* strain WWN1 and *Enterobacter cloacae* strain JWM6, respectively, and their consortium increased CAT activity by 46% as compared to the control. In case of 50 ppm, shoot CAT activity was recorded as 5.4–7 EU mg^–1^ protein. Plants inoculated with *Citrobacter werkmanii* strain WWN1 and *Enterobacter cloacae* strain JWM6 showed an increase of 26% and 25%, respectively, and the bacterial consortium increased CAT activity by 28% (7 EU mg^–1^ protein) compared to the respective control. Under 100 ppm HM stress, CAT activity ranged from 5 to 6.7 EU mg^–1^ protein. *Citrobacter werkmanii* strain WWN1 and *Enterobacter cloacae* strain JWM6 increased CAT activity by 30% and 26%, respectively, while when applied in consortium, an increase of 33% (6.7 EU mg^–1^ protein) was observed compared to uninoculated plants. At 200 ppm of HMs, the catalase activity was recorded in the range of 4–5.8 EU mg^–1^ protein. The maximum CAT activity was observed in plant shoots inoculated with the bacterial consortium, where an increase of 42% (5.8 EU mg^–1^ protein) was observed as compared to uninoculated plants.

Similarly, root CAT activity was also enhanced under HM stress. Without HM stress, root CAT activity was measured as 1.7–2.3 EU mg^–1^ protein. Plants inoculated with *Enterobacter cloacae* strain JWM6 showed a maximum increase of 31% (2.25 EU mg^–1^ protein) as compared to uninoculated plants. In case of 50 and 100 ppm HMs, root CAT activity ranged from 1.5 to 2.6 EU mg^–1^ protein and 1.02–2.7 EU mg^–1^ protein, respectively. The bacterial consortium showed the maximum increase in root CAT activity at both 50 and 100 ppm as compared to their respective controls. Under 200 ppm of HMs, the root CAT was observed between 0.76 and 1.93 EU mg^–1^ protein. The bacterial inoculation increased the root CAT activity, i.e., *Citrobacter werkmanii* strain WWN1 by 109%, *Enterobacter cloacae* strain JWM6 by 142%, and the bacterial consortium by 151% as compared to uninoculated plants (Figure 6C).

#### Ascorbate Peroxidase Activity

The APX content in plants significantly increased with the application of PGPB as compared to uninoculated plants. In wheat shoots, in the absence of HM stress, the APX content ranged from 0.56 to 2.11 EU mg^–1^ protein. A maximum increase of 277% (2.11 EU mg^–1^ protein) in shoot APX activity was observed in plants inoculated with *Citrobacter werkmanii* strain WWN1 as compared to the control. At 50 ppm HM stress, shoot APX activity ranged from 1.6 to 3.2 EU mg^–1^ protein. The bacterial consortium showed a maximum increase of 103% in shoot APX as compared to uninoculated plants. In case of 100 ppm HM stress, APX activity ranged from 1.16 to 2.95 EU mg^–1^ protein and *Citrobacter werkmanii* strain WWN1, *Enterobacter cloacae* strain JWM6, and their consortium increased shoot APX by 150, 153, and 152%, respectively, as compared to the respective control. At 200 ppm of HMs, the APX content ranged from 0.79 to 2.37 EU mg^–1^ protein. *Citrobacter werkmanii* strain WWN1 and *Enterobacter cloacae* strain JWM6 showed an increase of 199% (2.37 EU mg^–1^ protein) and 180%, respectively, and the bacterial consortium increased the shoot APX by 188%.

Like shoots, the bacterial inoculation significantly improved root APX activity both without and with HM stress. In plants without HM stress, root APX ranged from 0.94 to 1.85 EU mg^–1^ protein. The highest increase of 95% (1.8 EU mg^–1^ protein) was observed in plants inoculated with *Citrobacter werkmanii* strain WWN1. At 50 and 100 ppm of HMs, the APX content ranged from 0.47 to 1.53 EU mg^–1^ protein and 0.49 to 1 EU mg^–1^ protein, respectively. A maximum rise of 223 and 100% was observed at 50 and 100 ppm, respectively, in plant roots inoculated with the bacterial consortium. Under 200 ppm HM stress, APX was in the range of 0.32–0.80 EU mg^–1^ protein. *Enterobacter cloacae* strain JWM6 and *Citrobacter werkmanii* strain WWN1 increased root APX by 149% (0.8 EU mg^–1^ protein) and 135%, respectively, while the bacterial consortium–inoculated plants showed an increase of 98% as compared to uninoculated plants (Figure 6D).

### Heavy Metal Accumulation in Shoots and Roots of Wheat

Inoculation with PGPB significantly reduced the HM uptake in both the shoots and roots of wheat under all HM concentrations. Under 50 ppm of added Cd, Ni, and Pb, HM accumulation in plant shoot ranged from 8.13 to 13.20 ppm for Cd, 7.6 to 15.4 ppm for Pb, and 8.2 to 14.5 ppm for Ni. The lowest HM uptake was recorded when plants were inoculated with the bacterial consortium compared to sole application of the studied bacteria. The consortium reduced the uptake of Cd by 38%, Pb by 50%, and Ni by 49% in comparison to uninoculated plants. At 100 ppm, the HM content in shoots ranged from 10.1 to 18.4 ppm (Cd), 13.8 to 22 ppm (Pb), and 12.7 to 21 ppm (Ni). The maximum decline in shoot HM uptake was observed in plants inoculated with the bacterial consortium where a decrease of 42% for Cd, 37% for Pb, and 36% for Ni was observed in plants inoculated with the bacterial consortium when compared with uninoculated plants. Under 200 ppm, Cd in the range of 16.8–26.3 ppm, Pb in the range of 16.5–27.5 ppm, and Ni in the range of 15.5–26.5 ppm were recorded in plant shoots. The maximum uptake reduction for Cd (36%), Pb (40%), and Ni (37%) was detected in plants inoculated with the bacterial consortium as compared to uninoculated plants.

**FIGURE 7:**
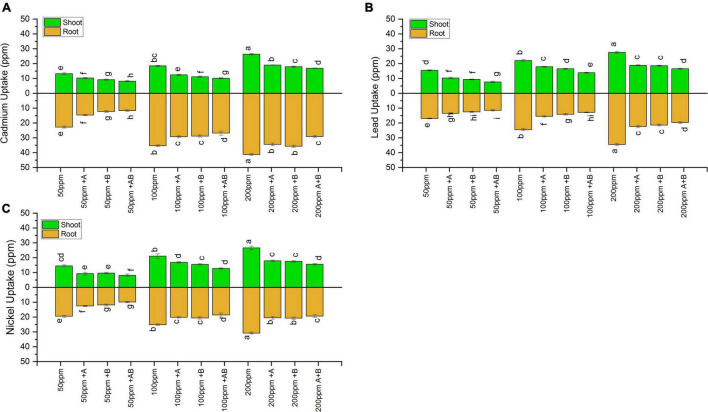
Heavy metal **(A)** cadmium, **(B)** lead, and **(C)** nickel uptake by shoots and roots of wheat (*Triticum aestivum* L.) grown under different concentrations of multi-HMs. Values are the mean of three replicates. Bars represent the standard error of means. Different letters above the bars indicate statistically significant difference between treatments at *P* ≤ 0.05. Details of treatments are the same as those in Figure 1.

Similar results were obtained for plant roots where HM accumulation increased with rising HM concentration. In roots, under 50 ppm HM concentration, HM uptake ranged from 11.6 to 22.7 ppm for Cd, from 11.5 to 16.9 ppm for Pb, and from 9.9 to 19.4 ppm for Ni. The bacterial consortium maximally reduced HM uptake, i.e., Cd by 48%, Pb by 32%, and Ni by 49%, as compared to uninoculated plants. Under 100 ppm, HMs in roots ranged from 26.8 to 35.4 ppm for Cd, 12.8 to 24.5 ppm for Pb, and 18.3 to 25.1 ppm for Ni. The maximum decrease in roots HM uptake was again observed in plants inoculated with the bacterial consortium where a decrease of 24% for Cd, 47% for Pb, and 29% for Ni was recorded compared to uninoculated plants. Under 200 ppm, the root HM concentration ranged from 29.1 to 41.2 ppm (Cd), 19.7 to 34.5 ppm (Pb), and 19.3 to 30.8 ppm (Ni). The maximum decrease for Cd (29%), Pb (42%), and Ni (29.2%) was observed in plant roots inoculated with the consortium as compared to uninoculated plants (Figure 7).

### Fourier Transform Infrared Spectroscopy Analysis of Bacterial Biomass

**FIGURE 8:**
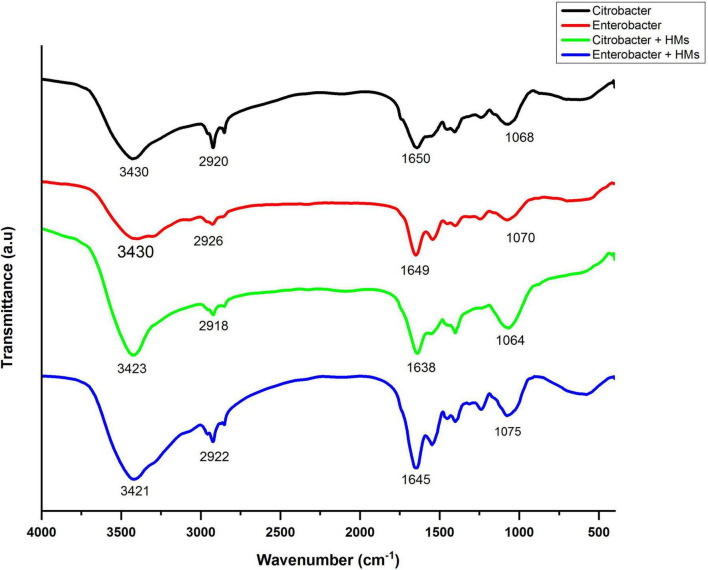
Infrared spectra of *Citrobacter werkmanii* strain WWN1 and *Enterobacter cloacae* strain JWM6 biomass in the presence of heavy metals (HMs) (Ni, Cd, Ni).

Fourier transform infrared spectroscopy spectra of bacterial biomass grown in the presence as well as absence of HMs were obtained in the range of 500–4000 cm^–1^. The FTIR spectrum of HM-loaded biomass displayed shifts in absorption peaks indicating interaction between HMs and bacterial biomass. The broad bands around 3430 cm^–1^ in control samples corresponded to alcohol/phenol O-H stretch and amine stretch. After treatment with HMs, these peaks shifted to 3423 cm^–1^ in the case of *Citrobacter werkmanii* strain WWN1 and to 3421 cm^–1^ in the case of *Enterobacter cloacae* strain JWM6 treated samples. These band peaks existed at a higher frequency in HM-loaded samples, indicating an increase in bond strength. The peaks at 2920 cm^–1^ and 2926 cm^–1^ in control samples corresponded to lipid–protein stretching. In HM-loaded samples, these peaks slightly shifted toward 2918 cm^–1^ and 2922 cm^–1^, respectively, indicating the interaction of lipid–protein with HMs. The 1650 cm^–1^ and 1649 cm^–1^ peaks in unloaded samples represented C-N stretching. These peaks shifted to 1638 cm^–1^ and 1645 cm^–1^ in HM-loaded samples, indicating interaction of these functional groups with HMs. Similarly, in control samples, peaks at 1068 cm^–1^ and 1070 cm^–1^ indicated protein amide (C = O) stretching, which shifted to 1064 cm^–1^ and 1075 cm^–1^ in HM-loaded samples (Figure 8).

### Pearson’s Correlation Coefficient (*r*) and Principal Component Analysis

**FIGURE 9:**
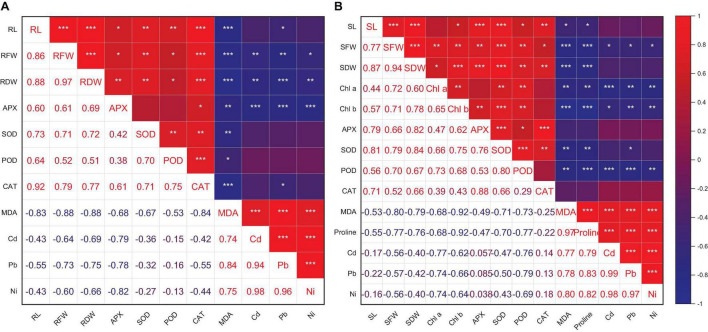
Pearson’s correlation coefficient (*r*) of growth attributes and antioxidant enzymes of wheat root **(A)** and shoot **(B)** with and without the inoculation of heavy metal–resistant plant growth–promoting bacteria under Cd, Ni, and Pb stress. RL (root length), RFW (root fresh weight), and RDW (shoot dry weight). SL, shoot length; SFW, shoot fresh weight; SDW, shoot dry weight; Chl a, chlorophyll a; Chl b, chlorophyll b. *p ≤ 0.05, **p ≤ 0.01, ***p ≤ 0.001.

**FIGURE 10:**
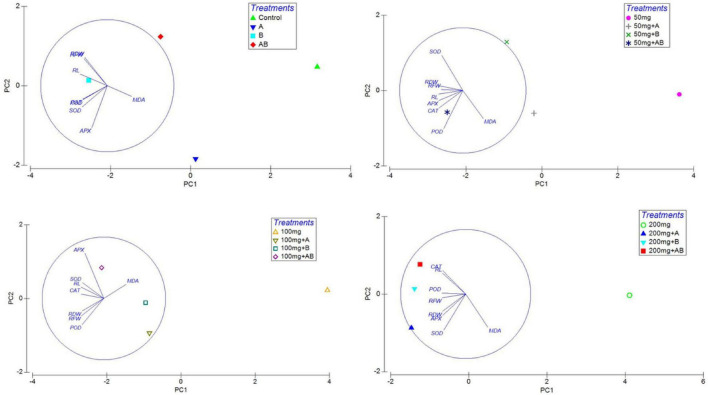
Principal coordinate analysis (PCA) of the effect of bacterial inoculation on RL, root length; RFW, root fresh weight; and RDW, root dry weight and antioxidants in wheat roots. Details of treatments are the same as those in Figure 1.

Heatmap responses of Pearson’s correlation coefficient (*r*) and PCA for the antioxidant enzymes, chlorophyll contents, stress determinants, and HM (Ni, Cd, and Pb) uptake by wheat shoots and roots under various HM concentrations are given in Figures 9, 10.

## Discussion

In the present study, we presented the beneficial role of seed inoculation with *Citrobacter werkmanii* strain WWN1 and *Enterobacter cloacae* strain JWM6 in wheat development and growth under HM stress. Siderophore production by soil bacteria such as *Citrobacter werkmanii* strain WWN1 and *Enterobacter cloacae* strain JWM6 (Ajmal et al., 2021) is a prerequisite for better nutrition and overall state of plants. Moreover, previous study revealed that both the isolates were positive for potassium, phosphate, and zinc solubilization assays *in vitro* (Ajmal et al., 2021). Resultantly, these strains might have had a positive effect on plant nutrition (Anand et al., 2016) because K, P, and Zn are very important nutrients in all metabolic processes of plants, including respiration, energy conversion, and photosynthesis. In the present study, bacterial inoculation either individually and in consortium significantly increased the growth and biomass of wheat possibly due to the production of siderophores and organic acids leading to nutrient availability to plants (Naveed et al., 2014; Shan et al., 2020; Han et al., 2021).

Contamination with HMs such as Cd, Ni, and Pb of soils results in iron deficiency in many plant species. It causes inhibition of both chloroplast and chlorophyll development, leading to leaves chlorosis (Rizwan et al., 2016). Moreover, HMs induce toxicity to thylakoid membrane integrity, which leads to inhibitions of enzymes like chlorophyll synthetase and Rubisco, causing chlorophyll degradation (Hashem, 2014). Microbial siderophores as released by *Citrobacter werkmanii* strain WWN1 and *Enterobacter cloacae* strain JWM6 also help plants in iron uptake, making them good candidates to prevent plant chlorosis due to HM stress. In our study, under HM stress, the consortium of *Citrobacter werkmanii* strain WWN1 and *Enterobacter cloacae* strain JWM6 improved the chlorophyll content of wheat probably due to iron uptake by bacterial siderophore–Fe complexes, resulting in higher chlorophyll content and biomass production of PGPB-inoculated plants. The positive impact of *Citrobacter werkmanii* strain WWN1 and *Enterobacter cloacae* strain JWM6 under HM stress was clearly evident from morphological and biochemical improvement of plants, i.e., root and shoot length, plant biomass, and chlorophyll content, due to successful root colonization of the studied bacterial strains in wheat. Similar to our study, *Serratia* sp. and *Pseudomonas* sp. were found to colonize in roots of wheat and rice as a signature of useful plant–microbe interaction under stress conditions (Khan and Singh, 2021; Pramanik et al., 2021). Our results are also in line with other reports, where *Enterobacter* sp. and *Citrobacter* sp. enhanced growth and improved chlorophyll content in rice by alleviating HM stress (Habib et al., 2016; Li et al., 2017; Maxton et al., 2018).

In addition to the abovementioned hazards, HMs also cause severe oxidative stress due to the release of excessive ROS in plants which induces changes in antioxidant enzyme activities. Antioxidant enzymes such as SOD, POD, APX, and CAT maintain the ROS at optimum levels (Ahmad et al., 2017). Elevated levels of ROS can cause severe disruption of physiological mechanisms in wheat and other plants, resulting in reduced growth and biomass (Mittler, 2002; Rizwan et al., 2016).

Among various antioxidants, SOD is a vital enzyme that converts superoxide into hydrogen peroxide (H_2_O_2_) at a high rate and protects cells against the harmful effects of ROS. Various HM stresses have been related to the alteration in SOD activities in different plants (Verma and Dubey, 2003). We also observed higher production of SOD in wheat plants grown in soil supplemented with HMs when inoculated with PGPB. In our study, SOD activity was more pronounced in roots under HM stress compared to that in the roots. This might be due to early accumulation and higher concentration of HMs in root tissues, thus inducing oxidative stress, and hence, SOD content might have been increased as a protective mechanism (AbdElgawad et al., 2020).

The next step in the ROS antioxidant process includes CAT oxidation that converts hydrogen peroxide (H_2_O_2_) into water (Noctor and Foyer, 1998). In the current study, CAT activity was also significantly increased in plants inoculated with *Citrobacter werkmanii* strain WWN1 and *Enterobacter cloacae* strain JWM6 under HM stress, which might have resulted in improved plant growth.

Similarly, POD activity was raised in plants inoculated with PGPB under HM stress. POD eliminates H_2_O_2_ by breaking it up into hydrogen and oxygen (Skórzyńska-Polit et al., 2003). POD is more efficient than CAT in scavenging H_2_O_2_ because of higher substrate affinity (Zhang et al., 2010). Therefore, if stress is not too severe for plant defense capacity, the major response is the release of POD and SOD rather than CAT. Previous studies have also reported higher, lower, or unchanged POD levels in response to HM stress depending upon the stress severity (Li et al., 2013; Hu et al., 2015; Gu and Liang, 2020). In our study, there was a significant increase in POD activity in *Citrobacter werkmanii* strain WWN1 and *Enterobacter cloacae* strain JWM6 inoculated wheat plants as compared to uninoculated plants. Moreover, POD facilitates lignin synthesis that can form a physical barrier against toxicity of HMs (Hegedüs et al., 2001). This also indicated that bacterial inoculation efficiently helped plants under HM stress by stimulating POD.

Generally, under HM stress, the APX content was higher in shoots than in roots and the APX content in bacteria-inoculated plants was significantly higher than the uninoculated plants. This suggests that the HM-tolerant PGPB used in the study aided plants to thrive better under HM stress. Our study showed that antioxidant enzyme activities dropped with an increase in HM concentration. This may be due to inhibition of enzymes as a result of elevated HM concentration. These findings are consistent with earlier reports (Alvarez and Lamb, 1997; Schickler and Caspi, 1999; Stroiński, 1999; Fatima and Ahmad, 2005; Awan et al., 2020).

Heavy metal stress, either directly or indirectly, is also responsible for molecular damage to plants due to the release of ROS. The increase in O_2_^–^ level might produce the hydroperoxyl radical, which converts various fatty acids to highly toxic lipid per oxides. An index of lipid peroxidation is routinely measured by the MDA content (Zhang et al., 2007). The level of MDA was significantly increased with increasing concentration of HM in soil. Moreover, the MDA content was higher in roots as compared to that in shoots, which might be because roots had stronger interaction with metals in soil. However, plants inoculated with *Citrobacter werkmanii* strain WWN1 and *Enterobacter cloacae* strain JWM6 or their consortium had a significantly lower MDA content as compared to uninoculated plants. This indicates that bacterial inoculation might have protected the plants from oxidative damage resulting from inactivation of the antioxidant enzyme system (Sgherri et al., 2007; Zhang et al., 2018).

Similarly, proline is one of the components of the non-specific defense systems toward HM toxicity and is known to play a key osmoregulatory role in plants (Saradhi, 1991). Proline is a molecular chaperone and has the ability to enhance the activity of different enzymes and protect protein integrity. In our study, *Citrobacter werkmanii* strain WWN1 and *Enterobacter cloacae* strain JWM6 treated plants showed lower proline content under HM stress in comparison to uninoculated plants. This implies that bacteria-inoculated plants may not have encountered higher HM stress and consequently lower proline accumulation was recorded (Gontia-Mishra et al., 2016). Inoculation with PGP bacteria decreased the proline content in leaves, which might have provided increased protection to plants (Sofy et al., 2020).

In addition to boosting the plant antioxidant enzyme defense system, seed inoculation with PGPB reduced the HM uptake by wheat compared with non-inoculated plants. The consortium of *Citrobacter werkmanii* strain WWN1 and *Enterobacter cloacae* strain JWM6 significantly reduced HM uptake in the shoots and roots of wheat as compared to single bacteria. Several other studies have also revealed that PGPB reduce the uptake of HMs in wheat shoots and roots growing in the presence of various HMs (Islam et al., 2014; El-Meihy et al., 2019; Ju et al., 2019; Saeed et al., 2019).

Finally, FTIR analysis of HM-tolerant *Citrobacter werkmanii* strain WWN1 and *Enterobacter cloacae* strain JWM6 grown in HM (Cd, Pb, and Ni) spiked liquid medium confirmed the presence of various moieties in bacteria, and shifts in the peaks indicated the binding of HMs with bacteria as explained by Das et al. (2007). In FTIR data, the shift in the peaks of hydroxyl functional groups in HM-loaded samples indicated the involvement of hydroxyl groups in binding the HMs. The shift in the peak appearing in the region attributed to alkyl chains (C-H stretching vibration) of fatty acids found in the phospholipid membrane (Ueshima et al., 2008) represented the involvement of these functional groups with HMs. The peak shift in the region attributed to amide suggests that these functional groups are also a major contributor to HM removal. In case of *Citrobacter werkmanii* strain WWN1 grown in the presence of HMs, the decreased intensity in fingerprint region peaks indicated the HM ion interaction with functional groups such as phosphates and proteins on the bacterial cell surface (Murthy et al., 2014). Similarly, the shift in the fingerprint region of HM-loaded samples represents the presence of carbon and phosphorus containing oxygen atoms interacting with HMs (Parikh and Chorover, 2006). Thus, various functional groups in the studied bacteria may be responsible for HM binding, therefore reducing the solubility and bioavailability and hence mitigating the toxicity of HMs to plants.

To sum up, multiple-HM stress severely affected the growth and physiology of wheat, whereas inoculation with HM-resistant PGP *Citrobacter werkmanii* strain WWN1 and *Enterobacter cloacae* strain JWM6 either alone or in consortium reduced Cd, Pb, and Ni uptake and toxicity and enhanced wheat growth. In general, the bacterial consortium performed better than individual bacterial strains in improving plant biomass and resistivity against the studied HMs.

## Conclusion

It is concluded that HM (Cd, Pb, and Ni) stress negatively impacted the growth, physiology, and antioxidant enzyme system of wheat. However, seed inoculation with PGP *Citrobacter werkmanii* strain WWN1 and *Enterobacter cloacae* strain JWM6 significantly improved plant growth; reduced HM accumulation in wheat shoots and roots; enhanced the production of antioxidants like SOD, POD, APX, and CAT; and lowered the MDA and proline contents in inoculated plants as compared to non-inoculated plants. Thus, seed inoculation with *Citrobacter werkmanii* strain WWN1 and *Enterobacter cloacae* strain JWM6 may be an effective method to enhance wheat production in HM-contaminated soils. However, further investigation is required to gain insight into the molecular mechanisms of HM detoxification and explore the impact of bacterial inoculation on biological activities of soil under varied environmental conditions.

## Data Availability Statement

The original contributions presented in the study are included in the article/Supplementary Material, further inquiries can be directed to the corresponding author/s.

## Author Contributions

AWA: experiment, data curation, software, methods, validation, and writing – original draft. HY: review and editing, methodolgy, resources, statistical analysis and supervision. MNH: review and editing, data curation, analysis, methododology, supervision, and resources. SM: orginal concept, funding acquisition, supervision, resources, methodolgy, software, and analysis. NK: review and editing, validation, resources. BLJ: review and editing, resources, and software.

## Conflict of Interest

The authors declare that the research was conducted in the absence of any commercial or financial relationships that could be construed as a potential conflict of interest.

## Publisher’s Note

All claims expressed in this article are solely those of the authors and do not necessarily represent those of their affiliated organizations, or those of the publisher, the editors and the reviewers. Any product that may be evaluated in this article, or claim that may be made by its manufacturer, is not guaranteed or endorsed by the publisher.
